# Microbiome mediation analysis: methods, assumptions, and practical considerations

**DOI:** 10.3389/fcimb.2026.1832981

**Published:** 2026-05-07

**Authors:** Ziman Jiang, Gen Li

**Affiliations:** Department of Biostatistics, University of Michigan, Ann Arbor, MI, United States

**Keywords:** compositional data analysis, counterfactual causal inference, Dirichlet regression, microbiome analysis, structural equation modeling

## Abstract

Microbiome mediation analysis provides a principled framework for understanding how environmental, behavioral, or clinical exposures influence human health through microbiomemediated biological pathways. However, its application is complicated by the compositional, sparse, and high-dimensional nature of microbiome data. A growing body of methods has been developed to address these challenges, drawing on structural equation modeling, counterfactual causal inference, distance-based testing, Bayesian variable selection, and nonparametric approaches. This paper reviews methodological developments designed to address these challenges and enable valid and interpretable mediation analysis in microbiome studies with particular emphasis on their underlying assumptions, limitations, and appropriate contexts of use, while also highlighting existing gaps and outlining future research directions.

## 1 Introduction

Analyses of microbiome data have substantially advanced our understanding of the complex relationships between the human body and microbial communities. Among the available statistical tools, mediation analysis provides a principled framework for elucidating the biological mechanisms through which environmental, behavioral, or clinical exposures influence health outcomes. In microbiome research, the microbiome can assume multiple roles: it may act as an exposure when the change in microbial composition may directly affect disease risk; as an outcome when external factors such as diet, medication, or environmental exposures alter microbial communities; or as a mediator that transmits the effects of upstream exposures to downstream health outcomes.

Among these settings, modeling the microbiome as a mediator is particularly challenging. Microbiome data are inherently high dimensional, sparse, and compositional, with complex dependence structures among taxa arising from ecological and evolutionary relationships. These characteristics complicate both statistical modeling and causal interpretation. Identifying which microbial taxa—mediate an exposure–outcome relationship therefore requires specialized methods that integrate compositional data analysis, regularization or dimension-reduction techniques, and causal inference principles. Despite these challenges, a growing body of evidence demonstrates that microbial taxa respond to a wide range of exposures and, in turn, influence host physiological processes and disease outcomes (Peterson et al., 2023). Consequently, mediation analysis with the microbiome as a mediator holds great promise for uncovering the biological pathways through which external factors exert their effects on human health.

**Figure 1:**
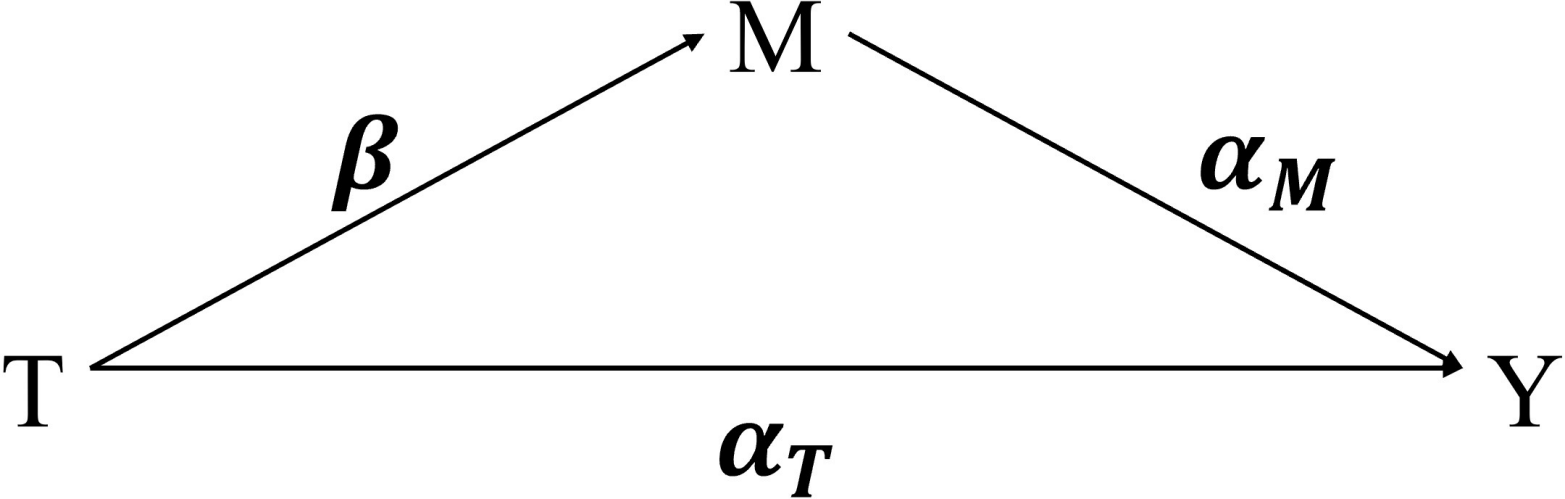
Diagram of mediation analysis. *T*: treatment; *M*: mediator; *Y*: outcome. The treatment *T* influences the mediator *M* with path coefficient *β_T_*, and the mediator *M* affects the outcome *Y* with coefficient *α_M_*, adjusting for *T*. The treatment *T* also directly affects the outcome *Y* with coefficient *α_T_*.

Formally, suppose there exists a causal relationship between an exposure or treatment *T* and an outcome *Y*, and let *M* denote a mediator such as the microbiome. Throughout this paper, the terms *exposure* and *treatment* are used interchangeably. Mediation analysis aims to evaluate whether, and to what extent, the effect of *T* on *Y* is transmitted through *M* (**Figure 1**). As illustrated in **Figure 1**, the exposure *T* influences the mediator *M* with path coefficient *β_T_*, and the mediator *M* in turn affects the outcome *Y* with coefficient *α_M_*, after adjusting for *T*. In addition, *T* may have a direct effect on *Y*, represented by the coefficient *α_T_*. The classical framework of Baron and Kenny (1986) formulates mediation through a set of linear regression models describing the treatment–mediator and mediator–outcome relationships. The total effect of *T* on *Y* is decomposed into a direct effect, captured by *α_T_*, and an indirect effect (mediation effect), defined as the product *β_T_*· *α_M_*, which quantifies the portion of the exposure effect transmitted through the mediator. In contrast, the counterfactual framework of VanderWeele (2016b) defines direct and indirect effects through contrasts between potential outcomes, providing a causal interpretation under some identification assumptions.

Many mediation methods have been developed to accommodate high-dimensional mediators, and they can be broadly grouped according to their modeling strategies (Zeng et al., 2021). Some approaches first perform dimension reduction or mediator selection to extract a lower-dimensional set of variables, and then apply standard mediation techniques to the resulting features (Zhang et al., 2018; Zhao et al., 2020; Huang and Pan, 2016). Other methods test mediation effects for individual mediators or small sets while accounting for the composite null hypothesis of no mediation (Liu et al., 2022b). A third class of approaches jointly models all potential mediators within a unified framework, adding assumptions on the exposure–mediator and mediator–outcome relationships to accommodate structural or sparsity constraints (Zhang et al., 2016; Song et al., 2020). However, in practice, many applied studies claiming microbiome-mediated mechanisms only rely on correlation-based or simple regression approaches that do not support causal mediation interpretations, highlighting a gap between commonly used analytical strategies and the assumptions required for valid inference (Tskhay et al., 2026). This discrepancy underscores the importance of carefully aligning methodological choices with their underlying assumptions when interpreting mediation effects.

However, unlike gene expression or proteomic data, microbial abundances are measured as relative abundances constrained to the simplex, such that an apparent increase in one taxon necessarily induces decreases in others, even in the absence of true biological changes (Gloor et al., 2017). Consequently, mediation methods developed for other high-dimensional omics data may produce spurious associations, biased effect estimates, or invalid inference if compositionality and interdependence are not properly addressed. Moreover, the combined effects of compositional constraints, high dimensionality, and zero inflation can substantially degrade the performance of traditional mediation methods (Blum et al., 2020). In addition, as noted by Hutkins et al. (2025), establishing causal links between microbiome modulation and health outcomes is difficult due to complex and unobservable mechanisms. Identifying which microbial taxa or functions are affected by the exposure such as prebiotics, is challenging due to interdependence and ecological interactions within microbial communities, and causal pathways often involve multiple taxa acting jointly rather than isolated effects. These challenges have motivated the development of specialized methodologies for microbiome mediation analysis that explicitly address compositional structure, sparsity, and complex dependence patterns among taxa (Peterson et al., 2023). The following sections review methodological developments that have been specifically designed to address these challenges and enable valid and interpretable mediation analysis in microbiome studies. **Table 1** provides a comparison of the microbiome mediation analysis methods discussed in this paper, and **Table 2** summarizes their publicly available implementations.

**Table 1:** Comparison of different mediation analysis methods for microbiome data.

Method	Mediator	Overall ME	Mediator-wise ME	Zero counts	Regularization	Model for mediator	Model for response
MedTest	PCoA	✓	×	–	×	LM	LM
MODIMA	Distance matrix	✓	×	–	×	LM	LM
IsometricLRTMM	ilr	×	✓	Pseudocount	de-biased Lasso	LM	LM
microHIMA	ilr	×	✓	Pseudocount	de-biased Lasso	Log-linear regression	LM
MarZIC	Composition	×	✓	–	×	Zero-inflated Beta regression	LM
CCMM	alr	✓	✓	Pseudocount	×	Compositional algebra	Linear log-contrast
SparseMCMM	alr	✓	✓	Pseudocount	Lasso	Dirichlet regression	Linear log-contrast
MicroBVS	ilr	✓	✓	Pseudocount	Bayesian priors	Log-linear regression	LM
PhyloMed	alr	✓	✓	Pseudocount	×	Log-linear regression	Log-linear regression
LDM	Composition	✓	✓	–	×	Inverse regression	Inverse regression
PERMANOVA-med	Distance matrix	✓	✓	–	×	Distance-based	Distance-based
NPEM	Entropy and mutual information	✓	✓	–	×	Information-based	Information-based
CRAmed	Composition	×	✓	–	×	ZINB	LM

SEM, structural equation modeling; ilr, isometric log-ratio transformation; alr, additive log-ratio transformation; LM, linear model; Taxa, raw taxonomic units used directly as mediators without compositional transformation; ZINB, zero-inflated negative-binomial model. Pseudocount refers to the technique of adding a small constant (usually 0.5) to zero counts to avoid mathematical issues in log-ratio transformations or other compositional analyses.

**Table 2:** Implementation in R for microbiome mediation methods.

Method	Implementation
MedTest	https://github.com/jchen1981/MedTest
MODIMA	https://cran.r-project.org/package=energy
PERMANOVA-med	https://github.com/yijuanhu/LDM
LDM	https://github.com/yijuanhu/LDM
SparseMCMM	https://github.com/chanw0/SparseMCMM
PhyloMed	https://github.com/tangzheng1/miMediation
microHIMA	https://github.com/joyfulstones/microbiome-mediation-SIS
IsometricLRTMM	Not publicly available
CCMM	https://cran.r-project.org/web/packages/ccmm/index.html
MarZIC	https://cran.r-project.org/web/packages/MarZIC/index.html
MicroBVS	https://github.com/mkoslovsky/MicroBVS
NPEM	https://github.com/anlingUA/NPEM
CRAmed	https://github.com/liudoubletian/CRAmed

## 2 Methods

Mediation analysis methods in microbiome research generally fall into two conceptual frameworks: the structural equation modeling (SEM) framework and the counterfactual framework. SEM-based approaches express the mediation process as a system of regression or path equations linking a treatment or exposure *T*, a mediator *M*, and an outcome *Y*, allowing estimation of indirect and direct effects through model coefficients as in the sets of Equations 1 and 2:

(1) $$\begin{array}{ll} M & =\beta_{T}T+{Є}_{1}, \end{array}$$

(2) $$Y=\alpha_{T}T+\alpha_{M}M+{Є}_{2}.$$

Early microbiome mediation studies adopted this framework by applying isometric log-ratio transformations (ilr) or summarizing community-level statistics to handle the compositional nature of microbial abundance data and fitting linear SEMs or mixed-effects path models (Zhang et al., 2018, 2021b). These approaches are intuitive and computationally straightforward, but they rely on assumptions of linearity, normality, and independent residuals that are often violated in high-dimensional, zero-inflated microbiome data (Wu et al., 2022).

In contrast, methods built on the counterfactual framework introduced by VanderWeele (2016b) define mediation effects causally through contrasts between hypothetical outcomes. Specifically, the Causal Indirect Effect (CIE) and the Causal Direct Effect (CDE) are derived by intervening on the exposure and mediator, thereby explicitly specifying potential outcomes for each unit under different treatments. For unit *i*, let *T_i_* denote a treatment or exposure variable, $M_{i}\in {\mathbb{R}}^{k}$ be a vector of mediators, *Y_i_* be an outcome. Let *M_i_*(*t*) be the potential mediator under *T_i_*= *t*, and *Y_i_*(*t,m*) the potential outcome under *T_i_*= *t* and *M_i_*= *m*. In addition, denote the observed variables as *M_i_*= *M_i_*(*T_i_*) and *Y_i_*= *Y_i_*(*T_i_,M_i_*(*T_i_*)). Based on these definitions, the CDE *ζ*(*τ*) and the CIE *δ*(*τ*) can be defined in Equation 3 to capture the effects of treatment on outcome through different pathways. Here, *t* and *t*_0_ represent different treatment conditions, *τ* denotes a specific treatment value, and *X_i_* denotes covariates.

(3) $$\begin{array}{ll} \zeta (\tau ) & =E[Y_{i}(t,M_{i}(\tau ))-Y_{i}(t_{0},M_{i}(\tau ))|X_{i}=x]\\ \delta (\tau ) & =E[Y_{i}(\tau ,M_{i}(t))-Y_{i}(\tau ,M_{i}(t_{0}))|X_{i}=x] \end{array}$$

The CDE captures the impact of changing the treatment from *T* = *t*_0_ to *T* = *t* on the outcome, while keeping the mediator *M* constant *M*(*τ*). In contrast, the CIE measures the effect of the mediator’s change from *M_i_*(*t*_0_) to *M_i_*(*t*) on the outcome, with the treatment *T* held constant *τ*.

Identification of causal mediation effects relies on several key assumptions: (1) consistency, which requires that the observed outcome corresponds to the potential outcome under the observed exposure and mediator values; (2) positivity, which requires that all levels of the exposure and mediator have positive probability given covariates; (3) sequential exchangeability, which assumes no unmeasured confounding for the exposure–outcome, exposure–mediator, and mediator–outcome relationships; (4) no mediator–outcome confounder affected by the exposure; and (5) cross-world independence, which assumes independence between counterfactual mediator and outcome under different exposure levels (VanderWeele, 2016a).

The counterfactual framework enables flexible quantification of mediation effects, even under nonlinear or nonparametric mediation models. The Causal Compositional Mediation Model (CCMM) introduced by Sohn and Li (2019) was among the first to explicitly integrate the counterfactual framework with compositional data theory. Subsequent methods have further built upon this framework (Wang et al., 2020; Wu et al., 2022). Overall, the counterfactual approach enables rigorous definitions of causal direct and indirect effects in the presence of compositional mediators. However, its applicability relies on strong causal assumptions, and its performance may be sensitive to zero inflation and data transformation choices commonly encountered in microbiome studies (Wang et al., 2020; Hutkins et al., 2025).

Regardless of the analytical framework used, a central goal of mediation analysis is to quantify and test the presence of mediation effects. In microbiome studies, these effects can be defined at different levels depending on the data structure and analytical strategy. Some approaches focus on community-level mediation, assessing whether the overall microbial community mediates the exposure–outcome relationship, while others target taxon-level mediation, aiming to identify specific taxa that contribute to the mediation effect. Accordingly, in the following subsections we group existing methods based on the level of mediation effects they address.

### 2.1 Community-level methods

OTU data are often extremely sparse; therefore, individual taxon-level tests tend to suffer from low statistical power. To enhance signal detection and reduce the multiple-testing burden, community-level approaches that evaluate all OTUs jointly have been proposed as a powerful alternative. For example, one may assess whether the association between dietary fiber intake and BMI is mediated by the gut microbiota through overall shifts in microbial community composition, as diet strongly influences human health in part through its effects on the gut microbiome (Wu et al., 2011). In such settings, community level methods can be used to detect an overall mediation effect without focusing on individual taxa. One strategy within this framework is to apply principal component analysis (PCA) to the microbiome data and use the resulting principal components (PCs) as aggregated microbial features for mediation testing (Zhang et al., 2018). MedTest, proposed by Zhang et al. (2018), represents one of the earliest distance-based methods of microbiome mediation analysis, treating the microbiome as a community-level mediator summarized by ecological distance measures (e.g., Bray–Curtis, UniFrac). By applying PCA to the distance matrix and using permutation-based inference, MedTest provides a flexible, nonparametric framework for detecting community-level mediation effects without explicitly modeling individual microbial taxa. Building on that idea, Hamidi et al. (2019) proposed MODIMA that generalized the distance-based paradigm by using distance/energy statistics to handle multivariate exposure, mediator and outcome simultaneously, producing an omnibus distance-correlation product test that broadened applicability to more complex multivariate settings.

However, these methods rely on implicit assumptions about how microbiome variation relates to exposure and outcome. In particular, MedTest assumes that the exposure–microbiome and microbiome–outcome associations are captured by the same set of principal components, which may not hold if different microbial features drive these relationships. In addition, both MedTest and MODIMA provide primarily global tests of association and do not directly quantify interpretable mediation effects, limiting mechanistic interpretation, and MODIMA does not adjust for confounding covariates. To address these limitations, Yue and Hu (2022a) proposed PERMANOVA-med, which extends permutational multivariate analysis of variance (PERMANOVA; Anderson, 2014) to the mediation setting. PERMANOVA tests associations between multivariate microbial community composition and covariates by partitioning sums of squares from a user-specified dissimilarity matrix (e.g., Bray–Curtis, UniFrac), with significance assessed by permutation. Building on this framework, PERMANOVA-med incorporates both exposure and outcome into a unified model and assesses mediation using an inverse-regression strategy similar to LDM-med (Yue and Hu, 2022b). The mediation effect in PERMANOVA-med is assessed through the product of PERMANOVA F-statistics, allowing covariate adjustment and improving robustness. However, interpretation remains at the community level, and linking detected effects to specific taxa or biological mechanisms remains challenging.

Mediation methods mentioned above primarily target community-level effects and generally ignore the underlying phylogenetic structure of the microbiome. PhyloMed (Hong et al., 2023) introduces a phylogeny-aware mediation framework that decomposes the microbial composition along the phylogenetic tree and conducts local mediation tests at internal nodes. By aggregating information from descendant taxa within each internal node, the method effectively pools weak or sparse mediation signals at the individual taxon level that are otherwise difficult to detect. This strategy yields both a community-level mediation test of microbiome and localized inference identifying mediating internal notes, substantially improving biological interpretability.

### 2.2 Taxon-level methods

Another line of research treats the relative abundances of all operational taxonomic units (OTUs) as mediators, conducts hypothesis testing at the individual-taxon level, and applies Bonferroni correction to control the family-wise error rate (FWER). For the dietary fiber–microbiome–BMI example, one may investigate whether dietary fiber influences BMI through specific microbial taxa, such as short-chain fatty acid–producing bacteria, which are known to mediate metabolic pathways (Makki et al., 2018). In this context, the goal is to identify which taxa drive the mediation effect. For instance, Zhang et al. (2021b) proposed isometricLRTMM, which uses the isometric log-ratio (ilr) transformation to handle compositionality and applies de-biased Lasso estimation to accommodate high-dimensional settings, followed by joint significance testing for mediation effects. The ilr transformation is as the following,


$${\tilde{M}}_{1}=\frac{1}{\sqrt{p(p-1)}}(\text{ln }\frac{M_{1}}{M_{2}}+\cdots +\text{ln }\frac{M_{1}}{M_{p}})=\sqrt{\frac{p-1}{p}}\text{ ln }\frac{M_{1}}{\sqrt[{p-1}]{\prod_{j=2}^{p}M_{j}}}\;,$$

where $\left(M_{1},\cdots ,M_{p}\right)$ is the relative abundance of *p* OTUs in a sample. The transformed variable ${\tilde{M}}_{1}$ retains all relative information about $M_{1}$ and reflects its contribution relative to the geometric mean of the remaining components. Thus, the product coefficients corresponding to ${\tilde{M}}_{1}$ represents a relative, rather than an absolute, mediation effect. In contrast, the interpretations of ${\tilde{M}}_{2},\ldots ,{\tilde{M}}_{p-1}$ are less direct because they do not involve $M_{1}$. To assess the mediation effect of a specific taxon l∈{2,…,p}, the authors therefore recommend reordering the composition so that $M_{l}$ becomes the first component, allowing its effect to be interpreted analogously to $M_{1}$. Building on a similar SEM-based framework with ilrtransformed relative abundances, Zhang et al. (2021a) further proposed microHIMA, which employs a closed testing–based selection procedure to control the FWER. Under the closed testing principle (Marcus et al., 1976), inference is conducted not on individual mediation hypotheses alone but on the entire family of corresponding intersection hypotheses. In practice, microHIMA evaluates mediation hypotheses in a hierarchical and structured sequence, thereby ensuring strong error control when simultaneously testing a large number of taxa.

IsometricLRTMM addresses compositionality through ilr transformations within a structural equation modeling framework; however, this comes at the cost of reduced interpretability and scalability. In particular, the approach assumes that the ilr transformation captures biologically meaningful contrasts and yields interpretable mediation effects, which may be challenging when multiple taxa jointly influence the outcome. Furthermore, the need for reordering complicates interpretation and comparison across taxa, limiting practical utility. In contrast, microHIMA employs a closed testing–based procedure to identify a subset of mediating taxa, but may be conservative, particularly when mediation effects are weak or distributed across many taxa.

Nonparametric Entropy Mediation (NPEM) by Carter et al. (2020), is a nonparametric-based method, which adopts the information-theoretic method for mediation analysis in high-dimensional metagenomic data. Rather than specifying parametric models for the treatment–mediator and mediator–outcome relationships, NPEM quantifies mediation using entropy and conditional mutual information by kernel density estimation, allowing it to capture nonlinear and complex dependency structures. It provided a univariate test to assess the effect of each taxon individually, alongside a bivariate test that combined information from both presence–absence and nonzero counts for each taxon. The bivariate test effectively accounts for the inherent sparsity of microbiome data.

In a similar vein, Wu et al. (2022) proposed MarZIC, which also addresses data sparsity by accounting for the large proportion of structural zeros arising from microbial absence. MarZIC extends counterfactual mediation analysis to zero-inflated compositional mediators by distinguishing mediation effects due to microbial presence–absence from those driven by changes in relative abundance. It adopts a marginal modeling strategy that separately links treatment to microbial prevalence and abundance, allowing the total mediation effect to be decomposed into presence-mediated and abundance-mediated components. Compositional constraints are handled using log-ratio transformations of nonzero components, and taxon level hypothesis tests are conducted to identify microbes mediating treatment effects through changes in prevalence or abundance.

In addition, Liu et al. (2025) proposed CRAmed, a randomization-based mediation method that uses a conditional randomization test (CRT) to assess mediation effect without relying on parametric model assumptions. CRAmed models the relationship using a zero-inflated negative binomial distribution and decomposes the natural indirect effect into presence–absence and abundance components. A joint significance test based on the zero-inflated version of distilled conditional randomization test (dCRT) introduced by Liu et al. (2022a) is used to identify significant mediators. The use of CRT enables the method to accommodate high-dimensional settings.

### 2.3 Other methods

CCMM (Sohn and Li, 2019) integrates counterfactual mediation analysis with compositional data theory to accommodate simplex-constrained mediators. To adjust the compositional constraints, CCMM adopts tools from compositional data analysis (Aitchison, 1982), including perturbation operators, power transformations, and additive log-ratio transformation. The treatment–mediator relationship is modeled on the simplex space. The mediator–outcome relationship is expressed through penalized log-contrast model proposed by Lin et al. (2014). This modeling strategy respects the relative nature of compositional data and avoids spurious correlations induced by unit-sum constraints. Inference in CCMM is conducted at both the community level and the taxon level. The total compositional mediation effect assesses whether the microbial composition as a whole mediates the treatment effect, while component-wise mediation tests target individual taxa or components. Hypothesis testing is performed using Sobel tests or a bootstrap approach based on the product of pathway coefficients, enabling identification of specific mediating components when the overall mediation effect is present.

Wang et al. (2020) proposed SparseMCMM by replacing the strictly algebraic compositional formulation of CCMM with a regression-based strategy that explicitly separates the mediator–outcome and treatment–mediator pathways. The treatment–mediator relationship was modeled using a Dirichlet regression framework, assuming microbial relative abundances follow a Dirichlet distribution, where the mean composition is linked to treatment and covariates through a log link. For the mediator–outcome pathway, the authors retain the log-contrast regression model used in CCMM. By selecting a reference taxon, microbial predictors are expressed as log-ratios of the remaining taxa relative to the reference, ensuring scale invariance. To guarantee model identifiability under the simplex constraint, both taxon-specific main effects and treatment–taxon interaction coefficients are constrained to sum to zero across taxa. The authors incorporated LASSO penalties in both the log-contrast outcome model and the Dirichlet regression handle high dimensionality. Within the counterfactual framework, they formally defined the average causal direct effect, mediation effect, and total effect. They further developed hypothesis tests based on permutation to assess mediation at both the community level and the taxon-specific level.

Yue and Hu (2022b) proposed LDM-med for microbiome mediation analysis as an extension of their earlier Linear Decomposition Model (LDM), an inverse-regression framework for assessing associations between microbiome taxa and covariates. Rather than modeling the treatment–mediator and mediator–outcome relationships separately, LDM-med evaluates mediation effects through a series of taxon-specific linear regression models that combine both relationships. They defined *T_r_* as the residual of *T* in Equation 1 and *Y_r_* as the residual of *Y* in Equation 2. In each model, the response is the *j*-th taxon *M_j_*, and the covariates consist of the residualized treatment *T_r_*, residualized outcome *Y_r_*. This one-model-per-taxon structure provides a direct mapping between taxa and regression models, enabling detection of mediation signals at the individual taxon. For community-level inference, LDM-med aggregates taxon-specific p values using the harmonic mean method (Wilson, 2019) and assesses significance through permutation to test the overall mediation effects.

Fu et al. (2023) proposed microBVS, a Bayesian framework for microbiome mediation analysis. The method extends the work by Koslovsky and Vannucci (2020), jointly models a binary treatment, high dimensional compositional microbial mediators, and an outcome within a unified Bayesian framework. MicroBVS also decomposes mediation analysis into two linked components. The treatment–mediator relationship is modeled using a Dirichlet–multinomial distribution to account for overdispersion in the count data, while the mediator–outcome relationship is modeled through linear regression on balances, the ilr-transformed relative abundances. Spike-and-slab priors enable selection of balances contributing to outcome variation, identifying microbial contrasts that mediate treatment effects. The fully Bayesian formulation allows simultaneous estimation of model parameters component-wise, mediation effects, and community-level mediation effects, with uncertainty quantified through posterior inference via Markov chain Monte Carlo sampling.

## 3 Discussion

Microbiome research presents a range of statistical, computational, and biological challenges that set it apart from conventional omics analyses. Microbiome data are composed of relative abundances constrained to sum to one, a feature that induces spurious correlations and violates the assumptions of standard statistical models unless explicitly addressed through compositional methods such as log-ratio transformations. Analytical complexity is further compounded by sparsity, as many taxa are absent or undetected in a substantial proportion of samples, resulting in excessive zeros that may arise from both biological and technical sources (Knight et al., 2018). Moreover, the number of microbial features often far exceeds the available sample size, leading to identifiability issues, overfitting, and unstable estimation in the absence of appropriate regularization or dimension-reduction techniques. In addition, microbial taxa are not independent but interact ecologically and share evolutionary history; failure to account for these dependencies or phylogenetic structure can reduce statistical power and distort inference.

These challenges are amplified in mediation analysis, where valid inference requires simultaneously addressing compositionality, sparsity, high dimensionality, and dependence while preserving the causal interpretation of mediation effects. Extending classical mediation frameworks from low-dimensional settings to high-dimensional microbiome data renders indirect effects unidentifiable without strong modeling assumptions, sparsity constraints, or dimension reduction. Existing methods span multiple statistical paradigms, including SEM, counterfactual causal inference, and nonparametric information theoretic frameworks, each offering distinct advantages and trade-offs in terms of robustness, interpretability, and inferential scope.

Several important directions remain to be explored in microbiome mediation analysis. First, more sophisticated modeling strategies are needed to better accommodate the compositional structure of microbiome data, as relative abundances are constrained to the simplex and induce complex dependence among taxa. For example, our previous work (Li et al., 2023) introduced a relative-shift modeling framework that directly models relative abundances from a relative-shift perspective. Extending causal mediation models under this relative-shift framework can account for compositional constraints without requiring transformations of relative abundance, thereby preserving interpretability. Second, incorporating advanced causal inference techniques could improve association detection by addressing complex effect modification and time-varying confounding, both of which are common in microbiome studies. In particular, the microbiome may serve as both a mediator and an effect modifier, representing distinct roles with different implications for interpretation and intervention (Bourdeau-Julien et al., 2023). Frameworks such as VanderWeele’s four-way decomposition (VanderWeele, 2014) provide a principled approach to disentangle mediation and interaction effects; however, most existing microbiome mediation methods do not explicitly accommodate this distinction. Time-varying confounders influenced by prior exposures in longitudinal settings can induce bias when handled with standard methods, while exposure effects may depend on baseline microbial composition, host characteristics, or ecological interactions (Keogh et al., 2018). Leveraging developments such as g-methods therefore has the potential to substantially enhance the robustness and interpretability of microbiome mediation analyses.

Third, more complex multi-omics mediation pathways warrant investigation by considering a multilevel mediation analysis (Tofighi and Thoemmes, 2014). The microbiome may represent only one component in a broader causal chain that also involves other omics modalities, such as gene expression or metabolic profiles. For instance, diet affects host physiology through both direct pathways and indirect pathways mediated by the microbiota and its metabolome (Perler et al., 2023). Finally, longitudinal mediation methods remain largely underdeveloped, despite the frequent collection of repeated microbiome and outcome measurements. For example, David et al. (2014) demonstrated pronounced temporal dynamics in healthy gut microbial communities. Ignoring such temporal dependence may obscure dynamic mediation mechanisms and weaken causal interpretation. Although several longitudinal differential abundance methods have been proposed (Paulson et al., 2017; Luo et al., 2017), approaches for longitudinal mediation analysis are still lacking.

Although recent work has focused on increasingly sophisticated models for microbiome mediation analysis, it remains unclear whether model complexity is the primary barrier to practical application. When the microbiome is viewed as a mediator, the central goal is to understand the mechanisms linking exposures to health outcomes. Ultimately, progress in this area may depend not only on methodological advances, but also on improving interpretability, clarifying assumptions, and strengthening study design to ensure valid and clinically meaningful inference.
